# Supplementary material for: Draft genome of the lined seahorse, Hippocampus erectus
Source: Gigascience. 2017 Apr 22;6(6):1–6. doi: 10.1093/gigascience/gix030 (PMC5459928; doi:10.1093/gigascience/gix030)
Supplement: GIGA-D-16-00137_Original_Submission.pdf [file gix030_GIGA-D-16-00137_Original_Submission.pdf]

Manuscript Click here to download Manuscript revised manuscript.docx

Data Note

Draft genome of the lined seahorse, *Hippocampus erectus*

Qiang Lin[1] §, Ying Qiu[2,3] §, Ruobo Gu[2,3,4] §, Meng Xu[5] §, Jia Li[3] §, Chao Bian[3,6,7] §, Huixian Zhang[1], Geng Qin[1], Yanhong Zhang[1], Wei Luo[1], Jieming Chen[3], Xinxin You[3,6], Mingjun Fan[3], Min Sun[3], Pao Xu[2,6], Byrappa Venkatesh[8], Junming Xu[3,4,6]*, Hongtuo Fu[2,6]*, Qiong Shi[3,4,6,9]*

[1]CAS Key Laboratory of Tropical Marine Bio-resources and Ecology, South China Sea Institute of Oceanology, Chinese Academy of Sciences, Guangzhou 510301, China

[2]Freshwater Fisheries Research Center, Chinese Academy of Fishery Sciences, Wuxi 214081, China

[3]Shenzhen Key Lab of Marine Genomics, Guangdong Provincial Key Lab of Molecular Breeding in Marine Economic Animals, BGI Academy of Marine Sciences, BGI Fisheries, BGI, Shenzhen 518083, China

[4]BGI Zhenjiang Institute of Hydrobiology, BGI Fisheries, Zhenjiang 212000, China.

[5]BGI-Shenzhen, BGI, Shenzhen 518083, China

[6]BGI Research Center for Aquatic Genomics, Chinese Academy of Fishery Sciences, Shenzhen 518083, China

[7]Centre of Reproduction, Development and Aging, Faculty of Health Sciences, University of Macau, Taipa, Macau, China

[8]Institute of Molecular and Cell Biology, A*STAR, Biopolis, 138673, Singapore

[9]Laboratory of Aquatic Genomics, College of Ecology and Evolution, School of Life Sciences, Sun Yat-Sen University, Guangzhou 510275, China

§ Contributed equally to this work.

*Correspondence: shiqiong@genomics.cn (QS), fuht@ffrc.cn (HF), xujunming@genomics.cn (JX)

Emails of all authors: linqiang@scsio.ac.cn (QL), qiuying@genomics.cn (YQ), guruobo@genomics.cn (RG), xumeng@genomics.cn (MX), lijia1@genomics.cn (JL), bianchao@genomics.cn (CB), qingeng@scsio.ac.cn (GQ), zhangyanhong@scsio.ac.cn (YZ), luowei3@scsio.ac.cn (WL), chengjieming@genomics.cn (JC), youxinxin@genomics.cn (XY), zhanghuixian@scsio.ac.cn (HZ), fanmingjun@genomics.cn (MF), sunmin@genomics.cn (MS), xup@ffrc.cn (PX), mcbbv@imcb.a-star.edu.sg (BV), xujunmin@genomics.cn (JX), fuht@ffrc.cn (HF), shiqiong@genomics.cn (QS)

Abstract

Background: Distributed mainly in tropical and temperate regions, seahorses appear more attractive because of their special body plan and male pregnancy. The lined seahorse, *Hippocampus erectus*, is an Atlantic species and mainly inhabits shallow sea-beds or coral reefs. It has become very popular in China for its wide use in traditional Chinese medicine; however, its biological data to support this research are extremely deficient. In order to improve the aquaculture yield of this valuable fish species, we are trying to develop genomic resources for assistant selection in genetic breeding. Here, we provide whole genome sequencing, assembling and gene annotation of the lined seahorse, which can be further applied for construction of a high-density genetic linkage map for its molecular breeding.

Findings: A total of 174.6-Gb (Gigabase) raw DNA sequences were generated by the Illumina Hiseq2500 platform. The final assembly of the lined seahorse genome is around 458 Mb, representing 94% of the estimated genome size (489 Mb by K-mer analysis). The contig N50 and scaffold N50 reached 14.57 kb and 1.97 Mb respectively. Quality of the assembled genome was assessed by BUSCO with prediction of 85% of the known vertebrate genes. Using homology-based and *de novo* annotation methods, we predicted 22,435 protein-coding genes in the generated assembly, which is similar to the reported gene number (23,458) from the tiger tail seahorse (*H. comes*). Further phylogenetic analyses revealed a significant neutral evolutionary rate and expansive existence of six *patristacin* genes in seahorses, which

may be closely related to their unusual male pregnancy.

Conclusion: We report a draft genome of the lined seahorse. These generated genomic data are going to support molecular breeding of this economically important fish, and also provide insights into the genetic mechanisms of its iconic morphology and male reproductive behavior.

Keywords: Genome, Assembly, Annotation, *Hippocampus erectus*

Data description

Background

Syngnathidae, an interesting teleost family, exhibits special morphological innovations and reproductive behavior, and these phenotypes have come into being through long-term molecular evolution [1, 2]. Seahorses (*Hippocampinae*) are popular and iconic species because of their unique body plan and male pregnancy. As a model organism, seahorses could provide exceptional clues for studying evolution in virtue of their closed brood pouch, male pregnancy and seasonal migration conflicting their small home range [3, 4]. Recently, we have reported the genome sequence of the tiger tail seahorse (*Hippocampus comes*) [5], and provided primary insights into the genetic basis of its iconic morphology. The genome also provided some novel insights in a number of areas, such as the *patristacin* subfamily of astacin metalloproteases that may be closely related to the unusual male pregnancy in the species, since they were expanded and highly expressed in the male brood pouch during mid- and late-pregnancy [5].

Here, we provided a draft genome of the lined seahorse (*H. erectus;* Figure 1), which inhabits in coastal waters in Western Atlantic such as Nova Scotia, Canada and northern Gulf of Mexico to Panama and Venezuela [6]. Similar to other seahorse species, the lined seahorse is lacking in biological data, and has been treated as vulnerable or endangered in the Red List of Threatened Species (IUCN, 2015) [7]. Its seasonal migration, population genetics and molecular adaptability are important research areas for the conservation of this fish species [8-10]. Moreover, the lined seahorse is easily domesticated for breeding, and it has become a popular and

commercially important ingredient for traditional Chinese medicine in China [11-15]. In order to study the evolutionary history of the lined seahorse and improve its aquaculture yield, we are trying to develop genomic resources for assistant selection in genetic breeding. Here, we performed whole genome sequencing, assembling and gene annotation of the lined seahorse, which can be further applied for construction of a high-density genetic linkage map for its molecular breeding.

Preparation and sequencing of DNA samples

Genomic DNA was extracted from a pool of four male lined seahorse (NCBI Taxonomy ID: 109281; Fishbase ID: 3283). All animal experiments were conformed to the guidelines of the Animal Ethics Committee and were approved by the Institutional Review Board on Bioethics and Biosafety of BGI (approval ID: FT16091). Seven libraries, including 3 short-insert libraries (200, 500 and 800 bp) and 4 long-insert libraries (2, 5, 10 and 20 kb) libraries, were constructed based on the standard protocol of Illumina (CA, USA) and sequenced using the Illumina HiSeq2500 platform. Finally, we generated a total of 174.6-Gb raw sequences.

Processing of the raw sequencing reads

The raw sequences contained some sequencing errors, which may reduce the quality of genome assembly. Hence we filtered these raw sequences with the following stringent filtering processes through SOAPfilter v2.2 software [16]: (1) Filtered reads with 40% low-quality bases (quality scores ≤ 7). (2) Removed reads with N bases more than 10%. (3) Trimmed reads with 5 low-quality bases at the 5’ end. (4) Discarded reads with adapter contamination and/or PCR duplicates. (5) Corrected raw reads from the short-insert libraries based on k-mer spectrum. Finally, we obtained 111.3-Gb of clean reads.

Estimation of the genome size and assembly of the genome sequences

The genome size was estimated based on k-mer spectrum [17] with the following formula: G = k-mer_number / k-mer_depth, where G is genome size, k-mer_number

is the total number of k-mer and k-mer_depth means the peak frequency that higher than any other frequencies. For the lined seahorse, the k-mer_number is 24,445,959,200 (based on 17-mer), and the k-mer_depth is 50. Therefore, the genome size was estimated to be approximately 489 Mb, which is much smaller than the estimation (695 Mb) of the tiger tail seahorse [5].

The generated clean reads were further assembled by SOAPdenovo2 (v2.04) [18] with optimized parameters (pregraph -K 27 -d 1; contig -M 1; scaff -b 1.5) to construct contigs and original scaffolds. Subsequently the gaps in the intra-scaffolds were filled using the reads of short-insert libraries by GapCloser1.12 [16]. Finally, the achieved total scaffold length reached up to 457,759,912 bp, which is smaller than that of the reported tiger tail seahorse (501,592,652 bp) [5]. The calculated scaffold N50 and contig N50 are 1.97 Mb and 14.57 kb respectively (Table 1), which are comparable with the values from the tiger tail seahorse [5] (see more details in Table 1).

Assessment of genome completeness

Benchmarking Universal Single-Copy Orthologs (BUSCO) [19] is a software application that can be used to evaluate the completeness of a genome assembly by genes selected from appropriate lineage-specific orthologous groups. For the lined seahorse, the analysis data proved that our assembly contains 73% complete and 12% partial of vertebrate BUSCO orthologues (3,023 genes in total), suggesting that our assembly is of high quality.

Repeat analysis

Tandem repeats were searched in the generated genome assembly by utilizing the Tandem Repeats Finder (v4.04) [20]. Transposable elements (TEs) were identified with an approach combined both homology-based and *de novo* predictions. First, RepeatMask (v3.3.0) [21] was employed to detect known TEs based on homologous search against the Repbase TE library (release 17.01) [22]. RepeatProteinMask (v3.3.0) [21], the updated software included in RepeatMasker package, was used to identify the TE relevant proteins. Subsequently, LTR_FINDER [23] and

RepeatModeler (v1.05) [24] were used with the default parameters to construct the *de novo* repeat library. Then we used RepeatMask [21] to identify and classify novel TEs against the *de novo* repeat library that was generated by LTR_FINDER [23] and RepeatModeler [24]. All repeats were finally combined together with filtering of those redundant repetitive sequences. In total, the lined seahorse genome comprises approximately 30.43% repetitive sequences, in which 28.12% are TEs. Interestingly, the most abundant type of TE is class II DNA transposon, which covered around 15% of the genome. Our data are similar to the report of the tiger tail seahorse [5], i.e., 24.82% are TEs with class II DNA transposon as the most abundant.

Gene annotation

***De novo* prediction:** The repetitive regions of genome sequences were replaced with 'N' to reduce the ratio of pseudogene annotations. Then we choose 1,000 full-length but randomly selected genes from zebrafish homology gene set to train the model parameters for AUGUSTUS. We subsequently employed AUGUSTUS3.0.1 [25] and GenScan1.0 [26] for *de novo* prediction of repeat-masked genome sequences. Short genes (less than 150 bp) and premature or frame-shifted genes were removed.

Homology-based annotation: Protein sequences of zebrafish (*Danio rerio*), medaka (*Oryzias latipes*), fugu (*Takifugu rubripes*), stickleback (*Gasterosteus aculeatus*) and Nile tilapia (*Oreochromis niloticus*) were downloaded from Ensembl (release 83) [27]. The protein sequences of the tiger tail seahorse (*H. comes*) were downloaded from our recently published genome data (Bioproject ID: PRJNA314292) [5]. Protein sets of these species were mapped to the assembled lined seahorse genome using TblastN (v2.2.19) [28] with E-value ≤ 1e-5. Genewise (v2.2.0) [29] was applied to refine the potential gene models of all alignments. Ultimately, we filtered short genes (less than 150 bp) and premature or frame-shifted genes.

Gene sets integration and optimization: The gene models based on *de novo* prediction and homology-based annotation were merged to form a comprehensive and non-redundant gene set using GLEAN [30]. Finally, we obtained a gene set containing 22,435 genes, which is similar to the reported gene number (23,458) of the tiger tail

seahorse [5].

Functional assignment

The protein sequences predicted from the lined seahorse genome were aligned to the Swiss-Prot and TrEMBL databases [31] using BlastP at E-value ≤ 1e-5. The motifs and domains were annotated using InterProScan [32] by searching publicly available databases including Pfam [33], ProDom [34], SMART [35], PRINTS [36] and PANTHER [37], and then retrieved Gene Ontology (GO) [38] annotation from the results of InterProScan. The gene pathways were assigned based on the best blast hit against KEGG database [39]. In summary, approximately 91% of the genes are supported by at least one related function from the searched databases (Swiss-Prot, Interpro, TrEMBL and KEGG).

Construction of gene families

Protein sequences of 7 ray-fin fishes, including zebrafish (*Danio rerio*), medaka (*Oryzias latipes*), fugu (*Takifugu rubripes*), stickleback (*Gasterosteus aculeatus*), Nile tilapia (*Oreochromis niloticus*), platyfish (*Xiphophorus maculatus*) and spotted gar (*Lepisosteus oculatus*), were downloaded from Ensembl (release 83) [27]. The protein sequences of the tiger tail seahorse (*H. comes*) were downloaded from our recently published genome data [5]. The consensus proteome set of the above 8 species and the lined seahorse were composed of a final dataset of 190,553 protein sequences. Finally, we used OrthoMCL [40] to cluster gene families and obtained 18,760 OrthoMCL families with all-to-all BLASTP strategy (E-value ≤ 1e-5) and a Markov Chain Clustering (MCL) default inflation parameter.

Phylogenetic analysis

We extracted 4,402 one-to-one orthologous genes from the above-mentioned gene family set. The protein sequences of each selected family were aligned using MUSCLE (v3.8.31) [41] with the default parameters. The protein alignments were then converted to corresponding coding sequences (CDS) using an in-house perl

script. All these translated CDS sequences were concatenated into a “supergene” for each species. The phase1 sites in the CDS sequences were also extracted and concatenated into a “supergene” using an in-house perl script. A phylogenetic tree was constructed using PhyML [42] based on the phase1 sequences (Figure 2). The bootstrap supporting value for the topology is 100. Our data demonstrate that the pairwise distance of the lined seahorse with the tiger tail seahorse was greater than that with any other teleosts, indicating that the neutral evolutionary rate of seahorses is significantly higher than that of any other teleosts [5].

Analysis of *patristacin* gene family

The *patristacin* subfamily of astacin metalloprotease family may be closely related to the unusual male pregnancy in seahorses, since we identified six *patristacin* genes in the tiger tail seahorse and confirmed their expansion and high expression in the male brood pouch [5]. We also analyzed this gene in the lined seahorse genome. We downloaded these protein sequences from the tiger tail seahorse genome and blasted them in the lined seahorse genome by manual validation. Definitely, we confirmed existence of the six *patristacin* genes in the lined seahorse (Figure 2).

Patristacin protein sequences of the two seahorses (the tiger tail seahorse and lined seahorse), platyfish, Nile tilapia and green spotted puffer were used for construction of a phylogenetic tree by MrBayes (v3.2). Our phylogenetic data demonstrate that the *patristacin* genes in the two seahorse species, as well as the platyfish, have expanded (Figure 3). As we know, the platyfish is a special species that fertilization and hatching of eggs occur within the maternal body (ovoviviparity). Our interesting data may suggest that the *patristacin* subfamily has evolved novel functions in ovoviviparity of the platyfish and male pregnancy of the seahorses [5].

Conclusion

Seahorse is a fascinating teleost group with special morphological innovations and reproductive behavior. In our previous genome paper about the tiger tail seahorse [5], we paid much attention to the genetic bases of its unique morphology and reproductive system. However, besides the spectacular aspects of the phenotype,

seahorse has been very popular for the traditional Chinese medicine with health promotion function. Here we report the first draft genome assembly of the lined seahorse, an economically important aquaculture fish in China. With availability of these genomic data, we can develop genetic markers for construction of a high-density genetic linkage map and subsequently for further genetic selection and molecular breeding. These works will support a significant increase of the aquaculture yield, which can produce remarkable economic benefits and realize the ecological protection of seahorses in the world. Our genome data will facilitate the genetic mechanism study and evolutionary history analysis of the lined seahorse.

Table 1 Comparison of genome assembly and annotation between the lined seahorse and the tiger tail seahorse

Genome assembly	Lined seahorse	Tiger tail seahorse
Contig N50 size (kb)	14.57	34.67
Scaffold N50 size (Mb)	1.97	1.87
Estimated genome size (Mb)	489	695
Assembled genome size(Mb)	457.76	501.59
Genome coverage (X)	243.05	192.05
Longest scaffold (bp)	7,855,128	9,810,584
Genome annotation		
Protein-coding gene number	22,435	23,458
Annotated functional gene number	20,427 (91.05%)	22,245 (94.83%)
Unannotated functional gene number	2,008 (8.95%)	1,213 (5.17%)
Transposable elements content	28.1%	24.8%

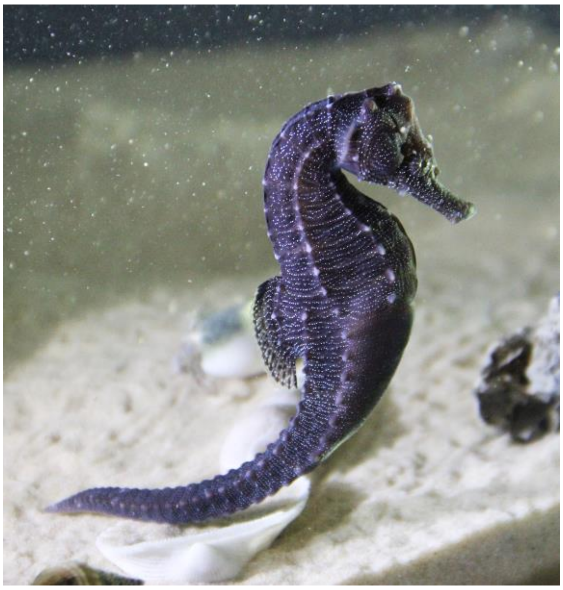

Figure 1 Photo of a cultivated line seahorse in Shenzhen, China.

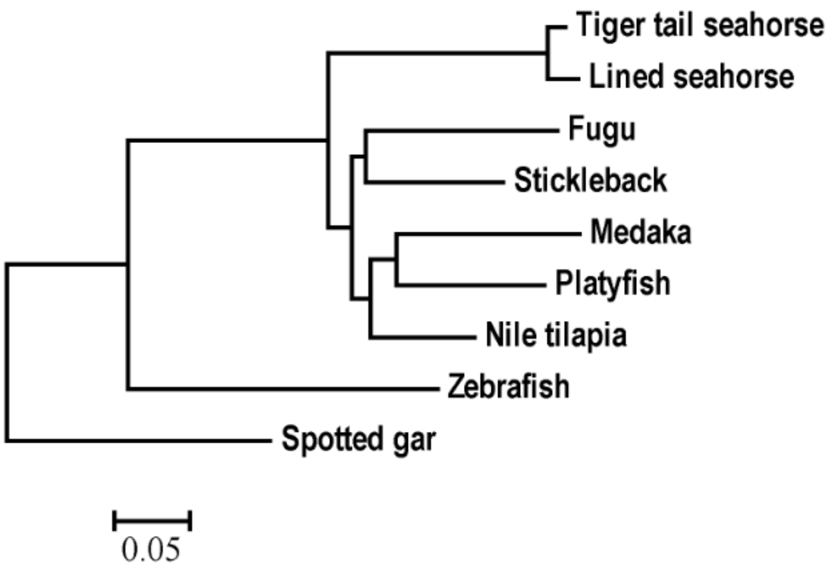

Figure 2 Phylogeny of ray-finned fishes. The Spotted gar was used as the outgroup species. See more details of the protein sequence sources in the main context.

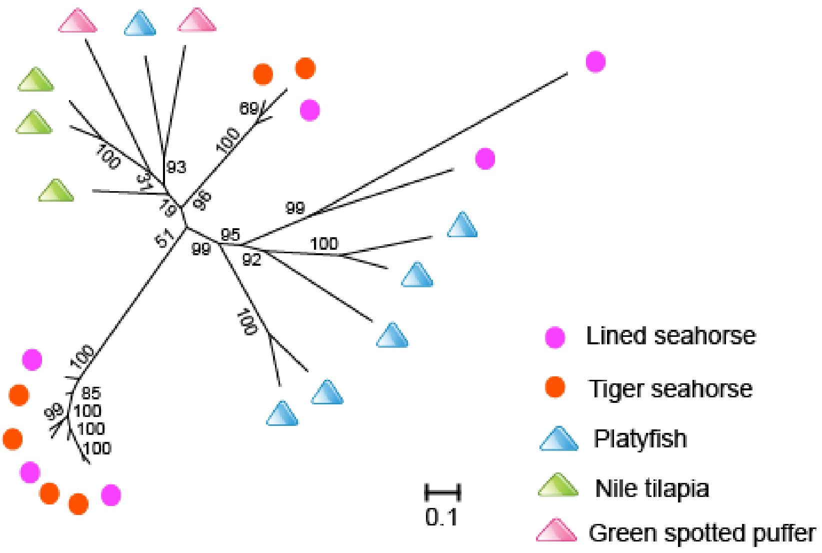

Figure 3 Phylogeny of *patristacin* genes in fishes. Note the remarkable expansion in seahorses and the platyfish.

Availability of supporting data

Supporting data are available in the GigaScience database and the raw data have been deposited in NCBI with the project accession PRJNA347499.

Author's contributions

QS and QL designed the project. JC, XY, MF and MS collected the samples and prepared the quality control. YQ, MX, JL, CB assembled and annotated the genome. YZ, HZ, GQ and WL were involved in the data analysis. YQ, QS, QL, PX and RG wrote the manuscript. JX, HF, BV and QS participated in discussions and provided advice. All authors read and approved the final manuscript.

Acknowledgements

This work was supported by the Youth Foundation of National High Technology Research and Development Program (2015AA020909), the Outstanding Youth Foundation in Guangdong Province (S2013050014802), the Special Fund for Agro-scientific Research in the Public Interest (201403008), the National Natural Science Foundation of China (41576145), China National Natural Science Foundation (No. 31370047), Shenzhen Special Program for Future Industrial Development (No. JSGG20141020113728803), Special Project on the Integration of Industry, Education and Research of Guangdong Province (No. 2013B090800017), Shenzhen Science and Technology Program (No. SGLH20131010105856414 & GJHZ20160229173052805), and Zhenjiang Leading Talent Program for Innovation and Entrepreneurship.

Competing interests

The authors declare that they have no competing interests.

References

1. A.B. Wilson, A. Vincent, I. Ahnesjo, *et al.*, **Male pregnancy in seahorses and pipefishes (family Syngnathidae): rapid diversification of paternal brood pouch morphology inferred from a molecular phylogeny**. *J Hered*, 2001. **92**(2): 159-166.
2. K.N. Stolting and A.B. Wilson, **Male pregnancy in seahorses and pipefish: beyond the mammalian model**. *Bioessays*, 2007. **29**(9): 884-896.
3. A. Harlin-Cognato, E.A. Hoffman, and A.G. Jones, **Gene cooption without duplication during the evolution of a male-pregnancy gene in pipefish**. *Proc Natl Acad Sci U S A*, 2006. **103**(51): 19407-19412.
4. S. Foster and A. Vincent, **Life history and ecology of seahorses: implications for conservation and management**. *Journal of fish biology*, 2004. **65**(1): 1-61.
5. Q. Lin, S. Fan, Y. Zhang, *et al.*, **The seahorse genome provides insights into**

the evolution of its iconic body plan and male pregnancy. *Nature*, 2016. in press.

6. F. Abe, H. Akimoto, A. Akopian, *et al*., **Observation of top quark production in p p collisions with the collider detector at fermilab**. *Physical review letters*, 1995. **74**(14): 2626.
7. P. Cardoso, P. Stoev, T. Georgiev, *et al*., **Species Conservation Profiles compliant with the IUCN Red List of Threatened Species**. *Biodivers Data J,* 2016(4): 1-3.
8. Q. Lin, J. Lin, and D. Zhang, **Breeding and juvenile culture of the lined seahorse, Hippocampus erectus Perry, 1810**. *Aquaculture*, 2008. **277**(3): 287-292.
9. Q. Lin, J. Lin, D. Zhang, *et al*., **Weaning of juvenile seahorses Hippocampus erectus Perry, 1810 from live to frozen food**. *Aquaculture*, 2009. **291**(3): 224-229.
10. W. Luo, H. Qu, J. Li, *et al*., **A novel method for the identification of seahorses (genus Hippocampus) using cross-species amplifiable microsatellites**. *Fisheries Research*, 2015. **172**: 318-324.
11. Q. Lin, D. Zhang, and J. Lin, **Effects of light intensity, stocking density, feeding frequency and salinity on the growth of sub-adult seahorses Hippocampus erectus Perry, 1810**. *Aquaculture*, 2009. **292**(1): 111-116.
12. Q. Lin, J. Lin, and L. Huang, **Effects of substrate color, light intensity and temperature on survival and skin color change of juvenile seahorses, Hippocampus erectus Perry, 1810**. *Aquaculture*, 2009. **298**(1): 157-161.
13. Q. Lin, W. Luo, S. Wan, *et al*., **De Novo Transcriptome Analysis of Two Seahorse Species (Hippocampus erectus and H. mohnikei) and the Development of Molecular Markers for Population Genetics**. *PloS one*, 2016. **11**(4): 1-19.
14. G. Qin, Y. Zhang, L. Huang, *et al*., **Effects of water current on swimming performance, ventilation frequency, and feeding behavior of young seahorses (Hippocampus erectus)**. *Journal of Experimental Marine Biology and Ecology*, 2014. **461**: 337-343.
15. X. Wang, Y. Zhang, G. Qin, *et al*., **A novel pathogenic bacteria (Vibrio fortis) causing enteritis in cultured seahorses, Hippocampus erectus Perry, 1810**. *Journal of fish diseases*, 2015.**39**:765-769.
16. R. Li, C. Yu, Y. Li, *et al*., **SOAP2: an improved ultrafast tool for short read alignment**. *Bioinformatics*, 2009. **25**(15): 1966-1967.
17. R. Li, W. Fan, G. Tian, *et al*., **The sequence and de novo assembly of the giant panda genome**. *Nature*, 2010. **463**(7279): 311-317.
18. R. Luo, B. Liu, Y. Xie, *et al*., **SOAPdenovo2: an empirically improved memory-efficient short-read de novo assembler**. *Gigascience*, 2012. **1**(1): 18.
19. F.A. Simao, R.M. Waterhouse, P. Ioannidis, *et al*., **BUSCO: assessing genome assembly and annotation completeness with single-copy orthologs**. *Bioinformatics*, 2015. **31**(19): 3210-3212.

20. G. Benson, **Tandem repeats finder: a program to analyze DNA sequences**. *Nucleic Acids Res*, 1999. **27**(2): 573-580.

21. M. Tarailo-Graovac and N. Chen, **Using RepeatMasker to identify repetitive elements in genomic sequences**. *Curr Protoc Bioinformatics*, 2009. **Chapter 4**: Unit 4 10.

22. J. Jurka, V.V. Kapitonov, A. Pavlicek, *et al.*, **Repbase Update, a database of eukaryotic repetitive elements**. *Cytogenet Genome Res*, 2005. **110**(1-4): 462-467.

23. Z. Xu and H. Wang, **LTR_FINDER: an efficient tool for the prediction of full-length LTR retrotransposons**. *Nucleic Acids Res*, 2007. **35**(Web Server issue): 265-268.

24. G. Abrusan, N. Grundmann, L. DeMester, *et al.*, **TEclass--a tool for automated classification of unknown eukaryotic transposable elements**. *Bioinformatics*, 2009. **25**(10): 1329-1330.

25. M. Stanke, O. Keller, I. Gunduz, *et al.*, **AUGUSTUS: ab initio prediction of alternative transcripts**. *Nucleic Acids Res*, 2006. **34**(Web Server issue): W435-439.

26. C. Burge and S. Karlin, **Prediction of complete gene structures in human genomic DNA**. *J Mol Biol*, 1997. **268**(1): 78-94.

27. F. Cunningham, M.R. Amode, D. Barrell, *et al.*, **Ensembl 2015**. *Nucleic Acids Res*, 2015. **43**(Database issue): D662-629.

28. D.W. Mount, **Using the Basic Local Alignment Search Tool (BLAST)**. *CSH Protoc*, 2007. **2007**.17.

29. E. Birney, M. Clamp, and R. Durbin, **GeneWise and Genomewise**. *Genome Res*, 2004. **14**(5): 988-995.

30. C.G. Elsik, A.J. Mackey, J.T. Reese, *et al.*, **Creating a honey bee consensus gene set**. *Genome Biol*, 2007. **8**(1): R13.

31. B. Boeckmann, A. Bairoch, R. Apweiler, *et al.*, **The SWISS-PROT protein knowledgebase and its supplement TrEMBL in 2003**. *Nucleic Acids Res*, 2003. **31**(1): 365-370.

32. S. Hunter, R. Apweiler, T.K. Attwood, *et al.*, **InterPro: the integrative protein signature database**. *Nucleic Acids Res*, 2009. **37**(Database issue): D211-215.

33. R.D. Finn, A. Bateman, J. Clements, *et al.*, **Pfam: the protein families database**. *Nucleic Acids Res*, 2014. **42**(Database issue): D222-230.

34. C. Bru, E. Courcelle, S. Carrere, *et al.*, **The ProDom database of protein domain families: more emphasis on 3D**. *Nucleic Acids Res*, 2005. **33**(Database issue): D212-215.

35. I. Letunic, R.R. Copley, S. Schmidt, *et al.*, **SMART 4.0: towards genomic data integration**. *Nucleic Acids Res*, 2004. **32**(Database issue): D142-144.

36. T.K. Attwood, **The PRINTS database: a resource for identification of protein families**. *Brief Bioinform*, 2002. **3**(3): 252-263.

37. P.D. Thomas, A. Kejariwal, M.J. Campbell, *et al.*, **PANTHER: a browsable database of gene products organized by biological function, using curated**

protein family and subfamily classification. *Nucleic Acids Res*, 2003. **31**(1): 334-341.

38. M. Ashburner, C.A. Ball, J.A. Blake, *et al*., **Gene ontology: tool for the unification of biology. The Gene Ontology Consortium**. *Nat Genet*, 2000. **25**(1): 25-29.
39. M. Kanehisa and S. Goto, **KEGG: kyoto encyclopedia of genes and genomes**. *Nucleic Acids Res*, 2000. **28**(1): 27-30.
40. L. Li, C.J. Stoeckert, Jr., and D.S. Roos, **OrthoMCL: identification of ortholog groups for eukaryotic genomes**. *Genome Res*, 2003. **13**(9): 2178-2189.
41. R.C. Edgar, **MUSCLE: multiple sequence alignment with high accuracy and high throughput**. *Nucleic acids research*, 2004. **32**(5): 1792-1797.
42. S. Guindon, J.-F. Dufayard, V. Lefort, *et al*., **New algorithms and methods to estimate maximum-likelihood phylogenies: assessing the performance of PhyML 3.0**. *Systematic biology*, 2010. **59**(3): 307-321.

 Click here to download Personal Cover Cover letter.docx

Dear Editor,

We would like to submit our manuscript, entitled "Draft genome of the lined seahorse, *Hippocampus erectus*", for publication as a Data Note in GigaScience. No any part of the entire manuscript has been published or has been accepted for publication elsewhere, and it is not being submitted to any other journal.

The lined seahorse, *Hippocampus erectus*, is an Atlantic species and mainly inhabits the shallow sea-beds or coral reefs. It has become very popular for traditional Chinese medicine in China, however, its biological data are extremely deficient. In order to improve the aquaculture yield of this valuable fish species, we are trying to develop genomic resources for assistant selection in genetic breeding.

Our manuscript is the first report of whole genome sequencing, assembling and gene annotation of the lined seahorse, which will support molecular breeding of this economically important fish, and also provide insights into the genetic mechanisms of its iconic morphology and male reproductive behaviors.

The manuscript includes 12 pages of text, 1 table and 1 figure, which were prepared according to your instructions to Authors.

We are looking forward to your reply at your earliest convenience.

Sincerely yours,

Qiong Shi, PhD, Professor
BGI
Shenzhen 518083
China
