# Supplementary material for: Draft genome of the lined seahorse, Hippocampus erectus
Source: Gigascience. 2017 Apr 22;6(6):1–6. doi: 10.1093/gigascience/gix030 (PMC5459928; doi:10.1093/gigascience/gix030)
Supplement: GIGA-D-16-00137_Revision_2.pdf [file gix030_GIGA-D-16-00137_Revision_2.pdf]

Manuscript
Click here to download Manuscript Hippocampus_ed_notrack_revised.docx

Data Note

Draft genome of the lined seahorse, *Hippocampus erectus*

Qiang Lin[1] §, Ying Qiu[2,3] §, Ruobo Gu[2,3,4] §, Meng Xu[5] §, Jia Li[3] §, Chao Bian[3,6,7] §, Huixian Zhang[1], Geng Qin[1], Yanhong Zhang[1], Wei Luo[1], Jieming Chen[3], Xinxin You[3,6], Mingjun Fan[3], Min Sun[3], Pao Xu[2,6], Byrappa Venkatesh[8], Junming Xu[3,4,6]*, Hongtuo Fu[2,6]*, Qiong Shi[3,4,6,9]*

[1]CAS Key Laboratory of Tropical Marine Bio-resources and Ecology, South China Sea Institute of Oceanology, Chinese Academy of Sciences, Guangzhou 510301, China

[2]Freshwater Fisheries Research Center, Chinese Academy of Fishery Sciences, Wuxi 214081, China

[3]Shenzhen Key Lab of Marine Genomics, Guangdong Provincial Key Lab of Molecular Breeding in Marine Economic Animals, BGI Academy of Marine Sciences, BGI Fisheries, BGI, Shenzhen 518083, China

[4]BGI Zhenjiang Institute of Hydrobiology, BGI Fisheries, Zhenjiang 212000, China

[5]BGI-Shenzhen, BGI, Shenzhen 518083, China

[6]BGI Research Center for Aquatic Genomics, Chinese Academy of Fishery Sciences, Shenzhen 518083, China

[7]Centre of Reproduction, Development and Aging, Faculty of Health Sciences, University of Macau, Taipa, Macau, China

[8]Institute of Molecular and Cell Biology, A*STAR, Biopolis, 138673, Singapore

[9]Laboratory of Aquatic Genomics, College of Ecology and Evolution, School of Life Sciences, Sun Yat-Sen University, Guangzhou 510275, China

§ Contributed equally to this work.

*Correspondence: shiqiong@genomics.cn (QS), fuht@ffrc.cn (HF), xujunming@genomics.cn (JX)

Emails of all authors: linqiang@scsio.ac.cn (QL), qiuying@genomics.cn (YQ), guruobo@genomics.cn (RG), xumeng@genomics.cn (MX), lijia1@genomics.cn (JL), bianchao@genomics.cn (CB), qingeng@scsio.ac.cn (GQ), zhangyanhong@scsio.ac.cn (YZ), luowei3@scsio.ac.cn (WL), chengjieming@genomics.cn (JC), youxinxin@genomics.cn (XY), zhanghuixian@scsio.ac.cn (HZ), fanmingjun@genomics.cn (MF), sunmin@genomics.cn (MS), xup@ffrc.cn (PX), mcbbv@imcb.a-star.edu.sg (BV), xujunmin@genomics.cn (JX), fuht@ffrc.cn (HF), shiqiong@genomics.cn (QS)

Abstract

Background: The lined seahorse, *Hippocampus erectus*, is an Atlantic species and mainly inhabits shallow sea-beds or coral reefs. It has become very popular in China for its wide use in traditional Chinese medicine. In order to improve the aquaculture yield of this valuable fish species, we are trying to develop genomic resources for assistant selection in genetic breeding. Here, we provide whole genome sequencing, assembly and gene annotation of the lined seahorse, which can enrich genome resource and further application for its molecular breeding.

Findings: A total of 174.6-Gb (Gigabase) raw DNA sequences were generated by the Illumina Hiseq2500 platform. The final assembly of the lined seahorse genome is around 458 Mb, representing 94% of the estimated genome size (489 Mb by k-mer analysis). The contig N50 and scaffold N50 reached 14.57 kb and 1.97 Mb respectively. Quality of the assembled genome was assessed by BUSCO with prediction of 85% of the known vertebrate genes and evaluated using the *de novo* assembled RNA-seq transcripts to prove a high mapping ratio (more than 99% transcripts could be mapped to the assembly). Using homology-based, *de novo* annotation and transcriptome-based prediction methods, we predicted 20,788 protein-coding genes in the generated assembly, which is similar to our previously reported gene number (23,458) of the tiger tail seahorse (*H. comes*).

Conclusion: We report a draft genome of the lined seahorse. These generated genomic data are going to enrich genome resource of this economically important fish,

and also provide insights into the genetic mechanisms of its iconic morphology and male pregnancy behavior.

Keywords: Genome, Assembly, Annotation, *Hippocampus erectus*

Data description

Background

Syngnathidae, an interesting teleost family, exhibit special morphological innovations and reproductive behavior, and these phenotypes have come into being through long-term molecular evolution [1, 2]. Seahorses (*Hippocampinae*) are popular and iconic species because of their unique body plan and male pregnancy. As an interesting model, seahorses could provide exceptional clues for studying evolution in virtue of their closed brood pouch, male pregnancy and seasonal migration [3, 4]. Recently, we have reported whole genome sequence of the tiger tail seahorse (*Hippocampus comes*) [5], and provided primary insights into the genetic basis of its iconic morphology. The work also dealt with a number of fascinating areas, such as the *patristacin* subfamily of astacin metalloproteases that may be closely related to the unusual male pregnancy in this species, since they were expanded and highly expressed in the male brood pouch during mid- and late-pregnancy [5].

Here, we provide a draft genome of the lined seahorse (*H. erectus*; Figure 1), which inhabits coastal waters in Western Atlantic such as Nova Scotia, Canada and northern Gulf of Mexico to Panama and Venezuela [6]. It has been treated as vulnerable or endangered in the Red List of Threatened Species (IUCN, 2015) [7]. Moreover, the lined seahorse is easily domesticated for breeding, and it has become a popular and commercially important ingredient for traditional Chinese medicine in China [8-12]. In order to study the evolutionary history of the lined seahorse and improve its aquaculture yield, we are trying to develop genomic resources for assisted selection in genetic breeding. Hence, we performed whole genome sequencing, assembly and gene annotation of the lined seahorse, which should facilitate further studies on species conservation and molecular breeding of this economically important fish.

Preparation and sequencing of DNA samples

Genomic DNA was extracted from a pool of four male lined seahorses (NCBI Taxonomy ID: 109281; Fishbase ID: 3283). All animal experiments were conformed to the guidelines of the Animal Ethics Committee and approved by the Institutional Review Board on Bioethics and Biosafety of BGI (approval ID: FT16091). Seven libraries, including 3 short-insert libraries (200, 500 and 800 bp) and 4 long-insert libraries (2, 5, 10 and 20 kb), were constructed based on the standard protocol of Illumina (CA, USA) and sequenced using the Illumina HiSeq2500 platform (the read length is 125 bp). Finally, we generated a total of 174.6-Gb raw sequences.

Processing of the raw sequencing reads

These raw sequences contained some sequencing errors, which may reduce the quality of genome assembly. Hence we filtered these raw sequences with the following stringent filtering processes through SOAPfilter (v2.2) software [13]: (1) Filtered reads with 40% low-quality bases (quality scores ≤ 7). (2) Removed reads with N bases more than 10%. (3) Trimmed reads with 5 low-quality bases at the 5' end. (4) Discarded reads with adapter contamination and/or PCR duplicates. (5) Corrected raw reads from the short-insert libraries based on k-mer spectrum. Finally, we obtained 111.3 Gb of clean reads in total, in which 12.0, 14.5, 13.7, 18.0, 15.9, 18.9 and 18.3 Gb were kept from the seven sequencing libraries (from 200 bp to 20 kb) respectively.

Estimation of the genome size and assembly of the genome sequences

The genome size was estimated based on k-mer spectrum [14] with the following formula: G=k-mer_number/k-mer_depth, where G is the genome size, k-mer_number is the total number of k-mer, and k-mer_depth means the peak frequency that higher than any other frequencies. For the lined seahorse, the k-mer_number is 24,445,959,200 (based on 17-mer), and the k-mer_depth is 50. Therefore, the genome size was estimated to be approximately 489 Mb, which is much smaller than our estimation (695 Mb) for the tiger tail seahorse [5].

The generated clean reads were further assembled by SOAPdenovo2 (v2.04) [15] with

optimized parameters (pregraph -K 27 -d 1; contig -M 1; scaff -b 1.5) to construct contigs and original scaffolds. Subsequently the gaps in the intra-scaffolds were filled using the reads of short-insert libraries by GapCloser1.12 [13]. Finally, the achieved total scaffold length reached up to 457,759,912 bp with 2.8% gaps (12.8 Mb), which is smaller than that of the reported tiger tail seahorse (501,592,652 bp) [5]. The calculated scaffold N50 and contig N50 are 1.97 Mb and 14.57 kb respectively (Table 1), which are comparable with the values from the tiger tail seahorse [5] (see more details about the comparison in Table 1).

Assessment of genome completeness

Benchmarking Universal Single-Copy Orthologs (BUSCO) [16] is a software that can be used to evaluate the completeness of a genome assembly by genes selected from appropriate lineage-specific orthologous groups. For the lined seahorse, the analysis data proved that our assembly contains 73% complete and 12% partial sequences of vertebrate BUSCO orthologues (3,023 genes in total).

Simultaneously completeness of the lined seahorse genome was also evaluated using the *de novo* assembled RNA-seq transcripts from different developmental stages of the lined seahorse (downloaded from our recent paper [5]) to map the lined seahorse genome assembly with Blat [17]. All the results showed that more than 99% of transcripts could be mapped to the assembly (Table 2), suggesting that our assembly is of high quality.

Repeat analysis

Tandem repeats were searched in the generated genome assembly by utilizing Tandem Repeats Finder (v4.04) [18]. Transposable elements (TEs) were identified with an approach combined both homology-based and *de novo* predictions. First, RepeatMask (v3.3.0) [19] was employed to detect known TEs based on homologous search against the Repbase TE library (release 17.01) [20]. RepeatProteinMask (v3.3.0) [19], an updated software included in the RepeatMasker package, was used to identify the TE relevant proteins. Subsequently, LTR_FINDER [21] and

RepeatModeler (v1.05) [22] were used with the default parameters to construct the *de novo* repeat library. Then we used RepeatMask [19] to identify and classify novel TEs against this *de novo* repeat library. All the repeats were finally combined together with filtering of those redundant repetitive sequences. In total, the lined seahorse genome comprises approximately 30.43% repetitive sequences, in which 28.12% are TEs. Interestingly, the most abundant type of TE is class II DNA transposon, which covered around 15% of the genome. Our data are similar to the report of the tiger tail seahorse [5], in which 24.82 % are TEs with class II DNA transposon as the most abundant.

Gene annotation

***De novo* prediction:** Repetitive regions in the genome sequence were replaced with 'N' to reduce the ratio of pseudogene annotations. Then we chose 1,000 full-length but randomly selected genes from zebrafish homology gene set to train the model parameters for AUGUSTUS. We subsequently employed AUGUSTUS3.0.1 [23] and GenScan1.0 [24] for *de novo* prediction of repeat-masked genome sequences. Short genes (less than 150 bp) and premature or frame-shifted genes were removed.

Homology-based annotation: Protein sequences of zebrafish (*Danio rerio*), medaka (*Oryzias latipes*), fugu (*Takifugu rubripes*), stickleback (*Gasterosteus aculeatus*) and Nile tilapia (*Oreochromis niloticus*) were downloaded from Ensembl (release 83) [25]. Protein sequences of the tiger tail seahorse (*H. comes*) were downloaded from our recently published genome data (Bioproject ID: PRJNA314292) [5]. Protein sets of these species were mapped to the assembled lined seahorse genome using tBlastn (v2.2.19) [26] with E-value ≤ 1e-5. Genewise (v2.2.0) [27] was applied to refine the potential gene models of all alignments. Ultimately, we filtered short genes (less than 150 bp) and premature or frame-shifted genes.

Transcriptome-based prediction: We downloaded the transcriptome data of the lined seahorse from our previous work [10]. The raw reads were mapped onto the genome using TopHat (v2.0) [28] with the default parameters and assembled into transcripts using Cufflinks [29].

Gene set integration and optimization: The gene models based on *de novo* prediction, homology-based annotation and transcriptome-based prediction were merged to form a comprehensive and non-redundant gene set using GLEAN [30]. Finally, we obtained a gene set containing 20,788 genes, which is less than the reported gene number (23,458) of the tiger tail seahorse [5].

Annotation of *patristacin* gene family

The *patristacin* subfamily of astacin metalloprotease family may be closely related to the unusual male pregnancy in seahorses, since we identified six *patristacin* genes in the tiger tail seahorse and confirmed their expansion and high expression in the male brood pouch [5]. We also analyzed *patristacin* in the lined seahorse genome. Related *patristacin* protein sequences were downloaded from the tiger tail seahorse genome data [5] and used for homology searches against the lined seahorse genome using tBlastn (v2.2.19) [26]. We chose alignments with coverage >50% and identity >50% and then used Genewise (v2.2.0) [27] to predict the gene structures. We also downloaded the RNA-seq data at pregnancy stage of male lined seahorse from our recently published paper [10] to confirm existence of the six *patristacin* genes in the lined seahorse. The RNA-seq reads were mapped by TopHat [28] and gene expression levels were measured by RPKM (Reads Per Kilobases per Millionreads). Finally, we observed that all the six *patristacin* genes were expressed during pregnancy in the male lined seahorse.

Functional assignment

The protein sequences predicted from the lined seahorse genome were aligned to the Swiss-Prot and TrEMBL databases [31] using BlastP at E-value ≤ 1e-5. The motifs and domains were annotated using InterProScan [32] by searching publicly available databases including Pfam [33], ProDom [34], SMART [35], PRINTS [36] and PANTHER [37], and then retrieved Gene Ontology (GO) [38] annotation from the results of InterProScan. The gene pathways were assigned based on the best blast hit against KEGG database [39]. In summary, approximately 90.32% of the genes are

supported by at least one related function from the searched databases (Swiss-Prot, Interpro, TrEMBL and KEGG).

Construction of gene families

Protein sequences of seven ray-fin fishes, including zebrafish, medaka, fugu, stickleback, Nile tilapia, platyfish (*Xiphophorus maculatus*) and spotted gar (*Lepisosteus oculatus*), were downloaded from Ensembl (release 83) [25]. Protein sequences of the tiger tail seahorse (*H. comes*) were downloaded from our recently published genome data [5]. Protein sequences of Gulf pipefish (*Sygnathus scovelli*) were downloaded from the Cresko Lab web server (http://creskolab.uoregon.edu) [40]. The consensus proteome set of the above nine species and the lined seahorse were composed of a final dataset of 209,747 protein sequences. Finally, we used OrthoMCL [41] to cluster gene families and obtained 19,053 OrthoMCL families with all-to-all BLASTP strategy (E-value ≤ 1e-5) and a Markov Chain Clustering (MCL) default inflation parameter.

Phylogenetic analysis

We extracted 2,812 one-to-one orthologous genes from the above-mentioned gene family set. The protein sequences of each selected family were aligned using MUSCLE (v3.8.31) [42] with the default parameters. The protein alignments were then converted to corresponding coding sequences (CDS) using an in-house Perl script. All these nucleotide sequences were concatenated into a supergene for each species, which were used to construct a phylogenetic tree using PhyML (Figure 2) [43] .

Conclusion

Seahorses are a fascinating teleost group with special morphological innovations and reproductive behavior. In our previous genome paper about the tiger tail seahorse [5], we paid much attention to the genetic bases of its unique morphology and reproductive system. However, besides the spectacular aspects of the phenotype, seahorses have been very popular for the traditional Chinese medicine. Here we report

the first draft genome assembly of the lined seahorse, an important economically aquaculture fish in China. With availability of these genomic data, we can develop genetic markers for construction of a high-density genetic linkage map and subsequently for further genetic selection and molecular breeding in the future. These works will support a significant increase of the aquaculture yield, which can produce remarkable economic benefits and realize the ecological protection of seahorses in the world. Our genome data will also facilitate the genetic mechanism study and evolutionary history analysis of the lined seahorse.

Table 1 Comparison of genome assembly and annotation between the lined seahorse and the reported tiger tail seahorse

Genome assembly	Lined seahorse	Tiger tail seahorse
Contig N50 size (kb)	14.57	34.67
Scaffold N50 size (Mb)	1.97	1.87
Estimated genome size (Mb)	489	695
Assembled genome size(Mb)	457.76	501.59
Genome coverage (×)	243.05	192.05
Longest scaffold (bp)	7,855,128	9,810,584
Genome annotation		
Protein-coding gene number	20,788	23,458
Annotated functional gene number	18776 (90. 32%)	22,245 (94.83%)
Unannotated functional gene number	2012 (9.68%)	1,213 (5.17%)
Transposable elements content	28.1%	24.8%

Table 2 Assessment of the completeness of the lined seahorse genome using transcriptome data

**Dataset**	**Number**	**Total Length (bp)**	**Base covered by Assembly (%)**	**Sequence covered by Assembly (%)**	**With >90% sequence in one Scaffold**		**With >50% sequence in one Scaffold**	
					**Number**	**Percent (%)**	**Number**	**Percent (%)**
All	71765	52877091	98.22	99.52	68292	95.16	71255	99.29
>200bp	71765	52877091	98.22	99.52	68292	95.16	71255	99.29
>500bp	29811	40111717	98.12	99.68	27902	93.60	29640	99.43
>1000bp	14780	29612539	97.92	99.70	13561	91.75	14686	99.36

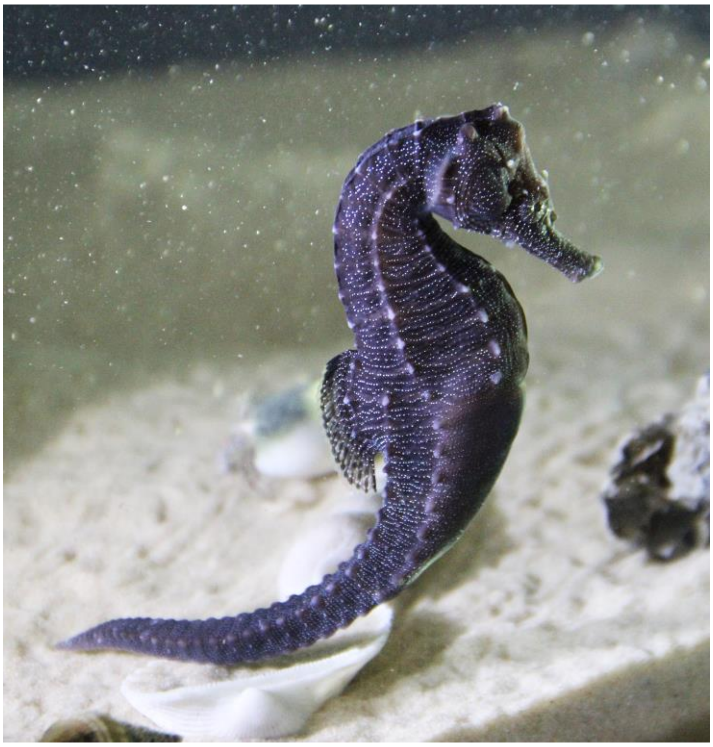

Figure 1 Photo of a cultivated line seahorse in Shenzhen, China.

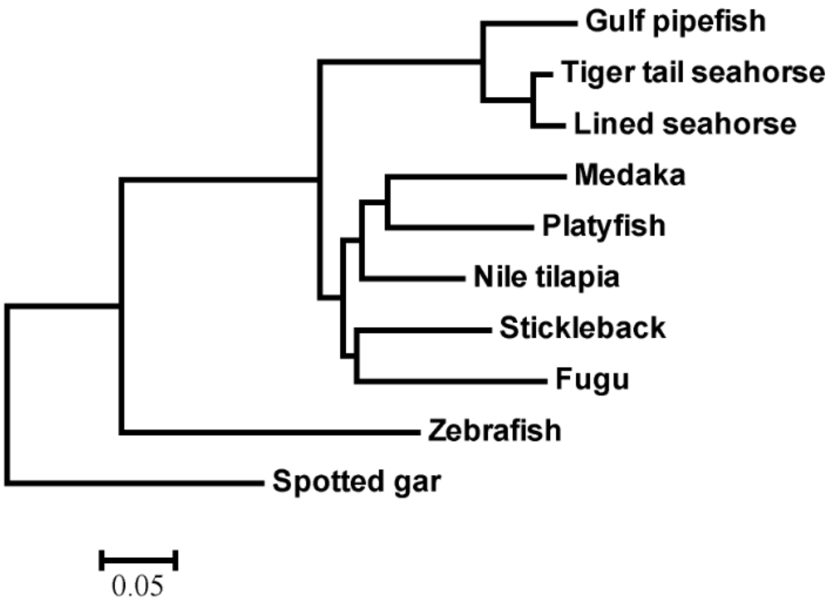

Figure 2 Phylogeny of ray-finned fishes. The Spotted gar was used as the outgroup species. See more details of the protein sequence sources in the main context.

Availability of supporting data

Supporting data are available in the *GigaScience* database [44] and the raw data have been deposited in NCBI with the project accession PRJNA347499.

Author's contributions

QS and QL designed the project. JC, XY, MF and MS collected the samples and prepared the quality control. YQ, MX, JL, CB assembled and annotated the genome. YZ, HZ, GQ and WL were involved in the data analysis. YQ, QS, CB, QL, PX and RG wrote the manuscript. JX, HF, BV and QS participated in discussions and provided advice. All authors read and approved the final manuscript.

Acknowledgements

This work was supported by the Youth Foundation of National High Technology Research and Development Program (2015AA020909), the Outstanding Youth Foundation in Guangdong Province (S2013050014802), the Special Fund for Agro-scientific Research in the Public Interest (201403008), the National Natural Science Foundation of China (41576145), China National Natural Science Foundation (No. 31370047), Shenzhen Special Program for Future Industrial Development (No. JSGG20141020113728803), Special Project on the Integration of Industry, Education and Research of Guangdong Province (No. 2013B090800017), Shenzhen Science and Technology Program (No. SGLH20131010105856414 & GJHZ20160229173052805), and Shenzhen Dapeng Special Program for Industrial Development (No. KY20160307).

Competing interests

The authors declare that they have no competing interests.

References

1. A.B. Wilson, A. Vincent, I. Ahnesjo, et al., **Male pregnancy in seahorses and pipefishes (family Syngnathidae): rapid diversification of paternal brood pouch morphology inferred from a molecular phylogeny**. *J Hered*, 2001. **92**(2): 159-66.
2. K.N. Stolting and A.B. Wilson, **Male pregnancy in seahorses and pipefish: beyond the mammalian model**. *Bioessays*, 2007. **29**(9): 884-96.
3. A. Harlin-Cognato, E.A. Hoffman, and A.G. Jones, **Gene cooption without duplication during the evolution of a male-pregnancy gene in pipefish**. *Proc Natl Acad Sci U S A*, 2006. **103**(51): 19407-12.
4. S. Foster and A. Vincent, **Life history and ecology of seahorses: implications for conservation and management**. *Journal of fish biology*, 2004. **65**(1): 1-61.
5. Q. Lin, S. Fan, Y. Zhang, et al., **The seahorse genome provides insights into the evolution of its iconic body plan and male pregnancy**. *Nature*, 2016.
6. F. Abe, H. Akimoto, A. Akopian, et al., **Observation of top quark production in p p collisions with the collider detector at fermilab**. *Physical review letters*, 1995. **74**(14): 2626.
7. P. Cardoso, P. Stoev, T. Georgiev, et al., **Species Conservation Profiles compliant with the IUCN Red List of Threatened Species**. *Biodivers Data J*, 2016(4): e10356.
8. Q. Lin, D. Zhang, and J. Lin, **Effects of light intensity, stocking density, feeding frequency and salinity on the growth of sub-adult seahorses Hippocampus erectus Perry, 1810**. *Aquaculture*, 2009. **292**(1): 111-116.
9. Q. Lin, J. Lin, and L. Huang, **Effects of substrate color, light intensity and temperature on survival and skin color change of juvenile seahorses, *Hippocampus erectus* Perry, 1810**. *Aquaculture*, 2009. **298**(1): 157-161.
10. Q. Lin, W. Luo, S. Wan, et al., **De Novo Transcriptome Analysis of Two Seahorse Species (*Hippocampus erectus* and *H. mohnikei*) and the Development of Molecular Markers for Population Genetics**. *PLoS One*, 2016. **11**(4): e0154096.
11. G. Qin, Y. Zhang, L. Huang, et al., **Effects of water current on swimming performance, ventilation frequency, and feeding behavior of young seahorses (*Hippocampus erectus*)**. *Journal of Experimental Marine Biology and Ecology*, 2014. **461**: 337-343.
12. X. Wang, Y. Zhang, G. Qin, et al., **A novel pathogenic bacteria (Vibrio fortis) causing enteritis in cultured seahorses, *Hippocampus erectus* Perry, 1810**. *Journal of fish diseases*, 2016, 39, 765-9.
13. R. Li, C. Yu, Y. Li, et al., **SOAP2: an improved ultrafast tool for short read alignment**. *Bioinformatics*, 2009. **25**(15): 1966-7.
14. B. Liu, Y. Shi, J. Yuan, et al., **Estimation of genomic characteristics by analyzing k-mer frequency in *de novo* genome projects**. *arXiv preprint arXiv:1308.2012*, 2013.
15. R. Luo, B. Liu, Y. Xie, et al., **SOAPdenovo2: an empirically improved memory-efficient short-read de novo assembler**. *Gigascience*, 2012. **1**(1): 18.
16. F.A. Simao, R.M. Waterhouse, P. Ioannidis, et al., **BUSCO: assessing genome assembly and annotation completeness with single-copy orthologs**. *Bioinformatics*, 2015. **31**(19): 3210-2.
17. W.J. Kent, **BLAT--the BLAST-like alignment tool**. *Genome Res*, 2002. **12**(4): 656-64.
18. G. Benson, **Tandem repeats finder: a program to analyze DNA sequences**. *Nucleic Acids Res*, 1999. **27**(2): 573-80.
19. M. Tarailo-Graovac and N. Chen, **Using RepeatMasker to identify repetitive elements in genomic sequences**. *Curr Protoc Bioinformatics*, 2009. **Chapter 4**: Unit 4 10.

20. J. Jurka, V.V. Kapitonov, A. Pavlicek, et al., **Repbase Update, a database of eukaryotic repetitive elements**. *Cytogenet Genome Res*, 2005. **110**(1-4): 462-7.
21. Z. Xu and H. Wang, **LTR_FINDER: an efficient tool for the prediction of full-length LTR retrotransposons**. *Nucleic Acids Res*, 2007. **35**(Web Server issue): W265-8.
22. G. Abrusan, N. Grundmann, L. DeMester, et al., **TEclass -- a tool for automated classification of unknown eukaryotic transposable elements**. *Bioinformatics*, 2009. **25**(10): 1329-30.
23. M. Stanke, O. Keller, I. Gunduz, et al., **AUGUSTUS: ab initio prediction of alternative transcripts**. *Nucleic Acids Res*, 2006. **34**(Web Server issue): W435-9.
24. C. Burge and S. Karlin, **Prediction of complete gene structures in human genomic DNA**. *J Mol Biol*, 1997. **268**(1): 78-94.
25. F. Cunningham, M.R. Amode, D. Barrell, et al., **Ensembl 2015**. *Nucleic Acids Res*, 2015. **43**(Database issue): D662-9.
26. D.W. Mount, **Using the Basic Local Alignment Search Tool (BLAST)**. *CSH Protoc*, 2007. **2007**: pdb top17.
27. E. Birney, M. Clamp, and R. Durbin, **GeneWise and Genomewise**. *Genome Res*, 2004. **14**(5): 988-95.
28. C. Trapnell, L. Pachter, and S.L. Salzberg, **TopHat: discovering splice junctions with RNA-Seq**. *Bioinformatics*, 2009. **25**(9): 1105-11.
29. C. Trapnell, B.A. Williams, G. Pertea, et al., **Transcript assembly and quantification by RNA-Seq reveals unannotated transcripts and isoform switching during cell differentiation**. *Nat Biotechnol*, 2010. **28**(5): 511-5.
30. C.G. Elsik, A.J. Mackey, J.T. Reese, et al., **Creating a honey bee consensus gene set**. *Genome Biol*, 2007. **8**(1): R13.
31. B. Boeckmann, A. Bairoch, R. Apweiler, et al., **The SWISS-PROT protein knowledgebase and its supplement TrEMBL in 2003**. *Nucleic Acids Res*, 2003. **31**(1): 365-70.
32. S. Hunter, R. Apweiler, T.K. Attwood, et al., **InterPro: the integrative protein signature database**. *Nucleic Acids Res*, 2009. **37**(Database issue): D211-5.
33. R.D. Finn, A. Bateman, J. Clements, et al., **Pfam: the protein families database**. *Nucleic Acids Res*, 2014. **42**(Database issue): D222-30.
34. C. Bru, E. Courcelle, S. Carrere, et al., **The ProDom database of protein domain families: more emphasis on 3D**. *Nucleic Acids Res*, 2005. **33**(Database issue): D212-5.
35. I. Letunic, R.R. Copley, S. Schmidt, et al., **SMART 4.0: towards genomic data integration**. *Nucleic Acids Res*, 2004. **32**(Database issue): D142-4.
36. T.K. Attwood, **The PRINTS database: a resource for identification of protein families**. *Brief Bioinform*, 2002. **3**(3): 252-63.
37. P.D. Thomas, A. Kejariwal, M.J. Campbell, et al., **PANTHER: a browsable database of gene products organized by biological function, using curated protein family and subfamily classification**. *Nucleic Acids Res*, 2003. **31**(1): 334-41.
38. M. Ashburner, C.A. Ball, J.A. Blake, et al., **Gene ontology: tool for the unification of biology. The Gene Ontology Consortium**. *Nat Genet*, 2000. **25**(1): 25-9.
39. M. Kanehisa and S. Goto, **KEGG: kyoto encyclopedia of genes and genomes**. *Nucleic Acids Res*, 2000. **28**(1): 27-30.
40. C.M. Small, S. Bassham, J. Catchen, et al., **The genome of the Gulf pipefish enables understanding of evolutionary innovations**. *Genome Biol*, 2016. **17**(1): 258.

41. L. Li, C.J. Stoeckert, Jr., and D.S. Roos, **OrthoMCL: identification of ortholog groups for eukaryotic genomes**. *Genome Res*, 2003. **13**(9): 2178-89.

42. R.C. Edgar, **MUSCLE: multiple sequence alignment with high accuracy and high throughput**. *Nucleic acids research*, 2004. **32**(5): 1792-1797.

43. S. Guindon, J.-F. Dufayard, V. Lefort, et al., **New algorithms and methods to estimate maximum-likelihood phylogenies: assessing the performance of PhyML 3.0**. *Systematic biology*, 2010. **59**(3): 307-321.

44. Lin Q, Qiu Y, Gu R, Xu M, Li J, Bian, C, et al. Supporting data for "Draft genome of the lined seahorse, Hippocampus erectus". *GigaScience* Database. 2017. http://dx.doi.org/10.5524/100298
