# Supplementary material for: Draft genome of the lined seahorse, Hippocampus erectus
Source: Gigascience. 2017 Apr 22;6(6):1–6. doi: 10.1093/gigascience/gix030 (PMC5459928; doi:10.1093/gigascience/gix030)
Supplement: Response_to_reviewer_comments_Original_Submission.pdf [file gix030_Response_to_reviewer_comments_Original_Submission.pdf]

Dear Hans,

Thanks for your instructions. We added related RNA-seq data, from our previous published papers (refs. 5 & 10), for genome and gene annotations. They are useful for evaluation of genome completeness and existence of six patristacin genes in the lined seahorse. Meanwhile, the phylogenetic analysis (Figure 2) was modulated according to the comments of the two reviewers.

We already uploaded our revised manuscript and the following point-by-point responses to the reviewers' comments into the journal system.

Your consideration at your earliest convenience is appreciated.

Best regards,

Qiong Shi, PhD, Professor
BGI
Shenzhen 518083
China

Reviewer reports:

Reviewer #1: This paper reports the genome sequence of the lined seahorse, Hippocampus erectus. The authors used a variety of established techniques to analyze and assemble short reads generated by Illumina HiSeq2500 for four male lined seahorses. The final assembly is 457.76 megabases in length, and the scaffold N50 is 1.97 megabases. Thus, future work will be required to assembly the scaffolds into linkage groups. The authors also annotated their scaffolds, estimating a total of 22,435 protein-coding genes. They investigated one gene of interest, patristacin, which has been shown to be involved in male pregnancy. This gene has undergone several recent gene duplication events in syngnathid fishes, and the lined seahorse genome contains six patristacin genes. The actual function of patristacin remains to be determined. Overall, this draft genome will be a valuable resource for scientists interested in the evolution and aquaculture of this interesting group of fish.

Specific comments:

(1) The first sentence of the abstract is unnecessary and should be removed.

Answer: Thanks for your suggestion. Removal of this sentence is done.

(2) Lines 75-76: I am not sure what the authors mean by "seasonal migration conflicting their small home range".

Answer: The lined seahorse migrates seasonally to deeper waters in the winters. In the revised manuscript, we changed the description to "seasonal migration" (line 72).

(3) Lines 215-218: The long branch leading to seahorses in the phylogeny cannot be interpreted due to the small number of species included in the phylogeny and the extremely large divergence times between seahorses and the closest outgroup. I think this sentence should be removed from the manuscript.

Answer: Thanks for your suggestion. This sentence was removed in the revised manuscript.

(4) The grammar should be carefully edited throughout the manuscript.

Answer: Thanks for your advice. Our manuscript was revised with help from an expert who has been in the America for over 9 years.

Reviewer #2: This paper describes the genome assembly and annotation of the lined seahorse, which is intended both as an auxiliary resource accompanying the recently published tiger tail seahorse (ref. 5), and as a first step to support genome-based breeding techniques for aquaculture. The assembly, annotation and validation meet the standards for an Illumina-based genome.

Some minor comments and issues I would like to ask the authors to address:

1) For completeness of the sequencing and assembly process, I would suggest reporting both the Illumina read lengths and the fraction of gaps in the final assembly (which, at 12.8 Mbp, is quite low).

Answer: Thank you for your good suggestions. The Illumina read lengths (125 bp) and the fraction of gaps in the final assembly (12.8 Mb) were added to the lines 100 and 126 respectively.

2) On genome size and completeness: the assembly size (458 Mbp) is close to the estimated genome size (489 Mbp), and close to the assembly size of the tiger tail seahorse (502 Mbp). The latter, however, is not close to its estimated size (695 Mbp, line 125), although it is relatively complete based on gene content. Both assemblies contain similar transposon landscapes, with no evidence for a recent burst in activity in the tiger tail seahorse (ref. 5). Therefore, the 695 Mbp estimate might be off? Although it does not impact the current paper, revisiting these estimates for both species might be worthwhile (perhaps using a more sophisticated model, e.g. qb.cshl.edu/genomescope).

Answer: Thanks for your comments. We applied the same k-mer analysis to predict genome sizes of the two seahorse species (as reported in ref. 5 and this manuscript). This method has been widely utilized for a majority of published genome papers, and the predicted results are always correct. Meanwhile, the classic flow cytometry also provided similar estimates (http://www.genomesize.com/result_species.php?id=3522).
By the way, the two genomes are remarkably different. For example, the tiger seahorse has a much higher proportion of repeat elements, which may lead to difficulty in genome assembly. Hence, we kept

the comparison to inspire deep investigations.

3) The BUSCO assessment of assembly completeness by itself does not suggest high quality (line 141), as >25% of orthologues appear to be missing or incomplete. And additional assessment of assembly completeness could establish high quality. For example, ref. 5 includes extensive RNA-seq from the lined seahorse. If these data align very well to the assembly, this would indicate that the BUSCO assessment is an underestimate.

Answer: Thanks for the question. In the revised manuscript, we quoted several de novo assembled RNA-seq datasets from different developmental stages of the lined seahorse (ref. 5), and mapped them onto the lined seahorse genome assembly using Blat. The achieved results showed that more than 99% of transcripts could be mapped to the assembly, suggesting that our assembly is of high quality. Please see more details in the revised Table 2 and lines 138-143.

4) On the phylogenetic analysis (lines 206-218): what are 'phase1 sites' (line 212)? I assume this is related to the initial supergene being constructed from translated CDSs, and the final analysis being done on DNA level. This should be explained more clearly.

Answer: Thanks for your suggestion. The phase1 sites mean the first nucleotide locus in a code and are each concatenated into a super gene using an in-house Perl script. Please see the explanation on lines 234-236 of the revised manuscript.

5) Also, the conclusion (lines 215-218) needs to be reformulated. Currently, it suggests that the distance *between* the seahorses is large, and that therefore they evolve faster than other teleosts (by neutral processes). I do not see this supported by figure 2. The seahorses are closely related, and yes the branch length to other teleosts is long, but this is best explained by the choice of sampled taxons (the seahorses are an early branching group amongst the Percomorpha).

Answer: Thanks for the advice. We removed this sentence in the revised manuscript.

6) On the patristacin genes (line 220-235 and figure 3): interestingly, both seahorse species have six copies. However, figure 3 clearly shows that they do not share all gene duplication (and gene loss) events. For example, the lined seahorse has two copies of the platyfish-like family, which are completely missing in the tiger tail seahorse. In other words (and slightly exaggerated), if the patristacin genes are responsible for or related to male brooding behaviour, then both seahorses evolved parts of this trait independently. (Please note that line 227 should also refer to figure 3 instead of figure 2).

Answer: You are right. The claim seems to be inappropriate. Hence, we only kept the annotation of patristacin genes for the lined seahorse and removed the comparison among different teleosts. Please see more details on lines 189-202 of the revised manuscript.

Reviewer #3: In Draft genome of the lined seahorse, Hippocampus erectus, Qiang Lin, et al. present the second seahorse genome and the third genome sequence from the family Syngnathidae for publication as a Data Note. The authors present a nice scaffold-level assembly that will surely be useful in the field. The biggest downside of the paper is that it sometimes reads like it is a supplement to the recently published Hippocampus comes genome from the same first author. The paper needs to provide more details as to some aspects of the assembly, and needs to discuss some of the shortcomings of the analysis (e.g. why was no RNA-seq data generated). Finally, several claims are made in the paper without evidence, apparently borrowed form the H. comes paper, which is inappropriate. These items are detailed below. Finally, the paper needs to be thoroughly copy edited to correct many small language errors.

Answer: Thanks for your criticism. In fact, the lined seahorse is very popular for the traditional Chinese medicine with health promotion function; simultaneously it's an economically important fish for aquaculture in China. Therefore, we sequenced the genome of the lined seahorse and provided the draft genome as a valuable genetic resource for studies on the evolution and aquaculture practices of this species. Apparently, it enriches genomic resource of the lined seahorse not just as a simple supplement to our recently published Hippocampus comes.
We also added the RNA-seq data of H. erectus that were previously published (ref. 5) for genome annotations. On the other hand, revision of our manuscript was performed with help from an expert who has been in the America for over 9 years.

*On line 86, the authors state that "the lined seahorse is lacking in biological data." This should be made more specific, as the authors cite papers, for example, on the development of transcriptome data for H. erectus, while the H. comes and S. scovelli genomes were just published.

Answer: Thanks for your comments on this section. You are right. Some genomes of the Syngnathidae were published recently (including our paper [ref 5]), and they provide valuable reference for our studies on the lined seahorse. Hence, we decided to delete the sentence in our revised manuscript.

*On line 88, the authors state that "population genetics and molecular adaptability are important research areas for the conservation of this fish species." Why are 'population genetics' and 'molecular adaptability' important?

Answer: Population genetics characterizes the extent of genetic variation within species and the reasons accounting for this variation. The objective of molecular adaptability is to study the molecular variation in the evolution. Therefore, we can conserve the lined seahorse when we know the evolution of the population genetics and molecular adaptability. However, we removed this sentence in the revised manuscript since it is misleading.

*In the abstract, the conclusion and on line 95 the authors state that the genome sequence will be further applied for the construction of a high-density genetic linkage map. However, no map is reported

in this paper and genome sequence is not required to generate a map. These statements make it appear as if a map has been developed.

Answer: Sorry for the confusion. We revised the statements in the abstract (lines 45 & 47) and on line 89, where we mentioned the new genome sequence will be helpful for “molecular breeding” or “assistant selection in genetic breeding”. Removal of the potential application for construction of a high-density genetic linkage map in the Abstract will eliminate the confusion, although the map is under construction by our collaborative teams.

*On lines 109-116, the authors discuss error in the are data and provide a list of programs that were applied to clean the data. However, the authors must discuss how many raw reads they started with, how many were discarded or trimmed and from which cleaning process. Further, how many reads contained adaptor sequence and how many were corrected using k-mers. If the numbers are considered abnormally high, the authors should discuss why they had high errors.

Answer: Thanks for your nice advice. Related information was added. Please see more details on lines 104-112.

*On lines 119-125 the authors discuss how they estimated genome size based on the k-mer spectrum of the raw data, citing the giant panda genome paper for their method. However, the formula they present does not match the formula in the giant panda genome paper. What is the methodological basis for their k-mer estimation method and why was a k-mer size of 17 used (instead of the k-mer size of 27 used for the assembly itself)?

Answer: Sorry for the mistake. In fact, the formula in our manuscript is a simplified version. We changed the reference 14 to “B. Liu, Y. Shi, J. Yuan, et al., Estimation of genomic characteristics by analyzing k-mer frequency in de novo genome projects. arXiv preprint arXiv:1308.2012” to introduce the derivation of the formula in detail. In de novo genome projects, analysis of the k-mer frequency has widely been used as an alternative way to estimate the genome sizes. A k-mer is a string extracted from reads with specified length k. When k=17, the k-mer theoretic number is 4^17 = 2^34 = 17G, which represents all possible k-mers. When assembling a genome, we increase the k-mer size to resolve repeats and obtain longer initial contigs; however, this will also increase the occupation of our computer resources. Usually, based on our previous experience, we take K-17 for a good balance. Please see more details on line 115-121 of the revised manuscript.

*On line 127 the authors state they used SOAPdenovo2 with 'optimized parameters'. How did the authors come to optimal values for these parameters and what do the parameters listed parenthetically mean?

Answer: Due to the parameters of Soapdenovo2 (especially the –K parameter) are empirical, we performed massive Soapdenovo analyses with a series of K parameters (25, 35, 45, 55, 65 and 75). After a careful evaluation, we selected the most optimized parameter -K 75 for further assembly. By the way,

this parameter -K 75 has been optimal for the genomic works of many reported species.

*On lines 168-176 the authors describe their gene annotation. Why was RNAseq data not used in the annotation (the authors cite a transcriptome for H. erectus)? What are the trade-offs of using H. comes genes to annotate versus using lined seahorse RNA? Would that bias your annotation, particularly with respect to patristacin genes?

Answer: Thanks for your nice advice. It's our negligence not to use RNA-seq data for gene annotation of the lined seahorse. Now, we downloaded corresponding transcriptome data of the lined seahorse from our previous report (ref. 10) and used them for our current annotation work. The gene annotation, functional assignment and phylogenetic analysis were modulated correspondingly in our revised manuscript. Existence of the six patristacin genes was also validated with these RNA-seq data. Please see more details on lines 197-200 of the revised manuscript.

*Line 212, what is a "phase1 site" with respect to the coding sequences?

Answer: The phase1 sites mean the first nucleotide locus in a code and are each concatenated into a super gene using an in-house Perl script. Please see the explanation on lines 234-236 of the revised manuscript.

*On line 216-218, the authors state that their tree indicates that "the neutral evolutionary rate of seahorses is significantly higher" than other teleosts. This claim is unsupported. The authors have not presented any data in this paper, based on the tree they constructed to support a claim on neutral evolutionary rates. (This claim originates in the H. comes paper, but the authors applied a number of analytical tests to their specific tree to support that claim.) This claim needs to be better supported with data or removed.

Answer: Thanks for the advice. We removed this sentence in the revised manuscript, although the statement is supported with more data in our recently published Nature paper (ref. 5).

*On lines 226-227, the authors state, "Definitely, we confirmed the existence of six patristacin genes." But how was this done, particularly without the availability of RNAseq data? No method is presented.

Answer: Thanks for your question. We supplemented detailed methods in the revised manuscript, and used RNA-seq data at pregnancy stage of male lined seahorse from our recently published paper (ref. 10) to confirm existence of the six patristacin genes in the lined seahorse. Please see more details on lines 189-202.

*For the species level analysis, the authors employed a maximum likelihood supergene approach with PhyML, but for the phylogenetic analysis of the patristacin genes the authors switch to a Bayesian framework with MrBayes. Why was this done and what method was employed in MrBayes? For example, why not use a partitioned gene-oriented approach with PhyML in this case? Some additional

description of what went into the phylogenetic analyses should be presented. How did you decide on the orthologous genes to include, why not include outgroup genes and outgroup species in the gene tree? Why not apply and compare both methods for the species and gene trees as is frequently done?

Answer: Thanks for your questions. In fact, we previously employed both PhyML and Bayesian framework to analyze the phylogeny of patristacin genes. As you mentioned above, the claim is not appropriate. Hence, we removed the claim and the phylogenetic tree (the previous Fig. 3) in our revised manuscript.

*The authors should consider including the S. scovelli orthologs in their species level analysis.

Answer: Thanks for your advice. The Sygnathus scovelli orthologs were added for the analysis. Please see more details on lines 220-221 and Figure 2 in the revised manuscript.

*On lines 228-235, the authors state the that the expansion of patristacin genes is related to platyfish and elements of male pregnancy. However, these claims are presented without any evidence, but simply a citation back to the H. comes genome paper. All the authors have presented is an unrooted tree showing a number of teleost patristacin genes grouped in different clades. The individual gene family members are not labeled and cannot be compared among the various orthologs and branches on the tree. This claim needs to be better supported with data or removed.

Answer: Thanks for your instructions and criticism. The claim is not appropriate with shortage of evidence, although we stated it for the tiger seahorse in our recent Nature paper (ref. 5). Hence, we only kept the annotation of patristacin genes for the lined seahorse and removed the comparison among different teleosts. Please see more details on lines 189-202 in the revised manuscript.

*The authors should consider including the patristacin genes from the newly published Sygnathus scovelli in their phylogenetic analysis.

Answer: Yes, it is done.

*Where can the assembled genome and annotation be obtained from? Only the raw data appear to have been deposited in NCBI.

Answer: The assembled genome, annotation and relevant Perl scripts were uploaded to the GigaScience Database. These data will be accessible once our paper is accepted for publication by GigaScience.

Minor errors:

Line 75-75, the meaning of "seasonal migration conflicting their small home range" is not clear.
Line 210, 213, "Perl" should be properly capitalized.
Line 225, "blasted" is not a verb.

Answer: Sorry for the mistakes. All these errors were revised as follows.
(1) It was changed to “seasonal migration”. Please find it on line 72 of the revised manuscript.
(2) The changes were done. See more details on lines 232 & 236.
(3) We changed it to “used for homology searches against … using tBlastn”. Please see more details on lines 194-195 of the revised manuscript.
