# Supplementary material for: Draft genome of the lined seahorse, Hippocampus erectus
Source: Gigascience. 2017 Apr 22;6(6):1–6. doi: 10.1093/gigascience/gix030 (PMC5459928; doi:10.1093/gigascience/gix030)
Supplement: Response_to_reviewer_comments_Revision_1.pdf [file gix030_Response_to_reviewer_comments_Revision_1.pdf]

Dear Hans,
Thanks for your kind help.
According to your suggestion, we removed “with health promotion functions” from the sentence you mentioned since no solid evidence is available. Please see more details on line 240 of the revised manuscript.
We also corrected certain minor errors based on the reviewers’ comments. Our revised manuscript and the following point-by-point responses to the reviewers’ comments were uploaded into your journal system.
Best regards,
Qiong Shi, PhD, Professor
BGI
Shenzhen 518083
China

Reviewer reports:

Reviewer #2: I am satisfied with most of the answers provided to the issues I raised. In particular, the RNA-seq evidence is useful and convincing. I do think the larger issue of completeness of this genome remains, but not necessarily in the technical sense (i.e. is the assembly of sufficient quality - it is) but in the comparative sense. For example, I do not really agree the number of genes reported is similar to that of the tiger tail seahorse (line 185). Also, I see no explicit support for the claim that the tiger tail seahorse contains a much higher proportion of repeat elements (in answer to my issue 2, on the genome size). But I agree that these issues could be part of a more in-depth comparative study.

That study should then also discuss the patristacin genes in more detail again, as that comparison was dropped from the current manuscript.

Answer: Thanks for your nice advice. Based on your suggestions, we changed the expression style with a more comparative sense. On lines 140-141 of our revised manuscript, the sentence was improved to be “suggesting that our assembly is of high quality”.
We changed ‘similar to’ to ‘less than’ for comparison of gene numbers. Please see the revision on line 184.
As you mentioned, we removed the discussion about high proportion of repeat elements (RE) in the tiger tail seahorse for a big genome size, since no solid evidence is available. We will perform in-deep investigations on RE and patristacin genes in a different study.

Some minor issues:
- I still think the 'phase 1' terminology is confusing (lines 233-237), and the current formulation states the same information twice (?). If correct, I would suggest replacing the entire section ['All these ... (Figure 2)'] with e.g. 'All these nucleotide sequences were concatenated into a supergene for each species, which were used to construct a phylogenetic tree using PhyML (Figure 2).'
- Figure 2 now includes a 'spooted gar'.

Answer: Sorry for the mistakes. You are right, hence we replaced the entire section ['All these ... (Figure 2)'] with 'All these nucleotide sequences were concatenated into a supergene for each

species, which were used to construct a phylogenetic tree using PhyML (Figure 2).' Please see more details on lines 232-234 of the revised manuscript. We also corrected the typo for “spotted gar” in Figure 2.

Reviewer #3: Ready for publication except for a few language issues:
Line 79 = 'provide'
Line 80 = 'inhabits coastal waters', delete 'in'
Line 87 = 'assisted'
Line 90 = delete 'were'
Line 136 = add 'sequences' after 'partial'

Answer: Thanks for your corrections. All these errors were revised as follows.
(1) It was done. Please find the revision on line 79 of the revised manuscript.
(2) We deleted ‘in’ as you mentioned on line 80.
(3) It was done for ‘assisted’ on line 86.
(4) The ‘were’ was deleted on line 94.
(5) The addition was done on line 134.
