# Supplementary material for: Draft genome of the lined seahorse, Hippocampus erectus
Source: Gigascience. 2017 Apr 22;6(6):1–6. doi: 10.1093/gigascience/gix030 (PMC5459928; doi:10.1093/gigascience/gix030)
Supplement: Reviewer_1_Report_(Original_Submission).pdf [file gix030_Reviewer_1_Report_(Original_Submission).pdf]

Reviewer Report

Title: "Draft genome of the lined seahorse, Hippocampus erectus"

Version: Original Submission **Date:** 12/6/2016

Reviewer name: Adam G. Jones

Reviewer Comments to Author:

This paper reports the genome sequence of the lined seahorse, Hippocampus erectus. The authors used a variety of established techniques to analyze and assemble short reads generated by Illumina HiSeq2500 for four male lined seahorses. The final assembly is 457.76 megabases in length, and the scaffold N50 is 1.97 megabases. Thus, future work will be required to assembly the scaffolds into linkage groups. The authors also annotated their scaffolds, estimating a total of 22,435 protein-coding genes. They investigated one gene of interest, patristacin, which has been shown to be involved in male pregnancy. This gene has undergone several recent gene duplication events in syngnathid fishes, and the lined seahorse genome contains six patristacin genes. The actual function of patristacin remains to be determined. Overall, this draft genome will be a valuable resource for scientists interested in the evolution and aquaculture of this interesting group of fish.

Specific comments:

(1) The first sentence of the abstract is unnecessary and should be removed.

(2) Lines 75-76: I am not sure what the authors mean by "seasonal migration conflicting their small home range".

(3) Lines 215-218: The long branch leading to seahorses in the phylogeny cannot be interpreted due to the small number of species included in the phylogeny and the extremely large divergence times between seahorses and the closest outgroup. I think this sentence should be removed from the manuscript.

(4) The grammar should be carefully edited throughout the manuscript

Level of Interest

Please indicate how interesting you found the manuscript: An article of importance in its field

Quality of Written English

Please indicate the quality of language in the manuscript: Needs some language corrections before being published

Declaration of Competing Interests

Please complete a declaration of competing interests, considering the following questions:

- Have you in the past five years received reimbursements, fees, funding, or salary from an organisation that may in any way gain or lose financially from the publication of this manuscript, either now or in the future?
- Do you hold any stocks or shares in an organisation that may in any way gain or lose financially from the publication of this manuscript, either now or in the future?
- Do you hold or are you currently applying for any patents relating to the content of the manuscript?
- Have you received reimbursements, fees, funding, or salary from an organization that holds or has applied for patents relating to the content of the manuscript?
- Do you have any other financial competing interests?
- Do you have any non-financial competing interests in relation to this paper?

If you can answer no to all of the above, write 'I declare that I have no competing interests' below. If your reply is yes to any, please give details below.

I declare that I have no competing interests.

I agree to the open peer review policy of the journal. I understand that my name will be included on my report to the authors and, if the manuscript is accepted for publication, my named report including any attachments I upload will be posted on the website along with the authors' responses. I agree for my report to be made available under an Open Access Creative Commons CC-BY license (http://creativecommons.org/licenses/by/4.0/). I understand that any comments which I do not wish to be included in my named report can be included as confidential comments to the editors, which will not be published.

I agree to the open peer review policy of the journal
