# Supplementary material for: Draft genome of the lined seahorse, Hippocampus erectus
Source: Gigascience. 2017 Apr 22;6(6):1–6. doi: 10.1093/gigascience/gix030 (PMC5459928; doi:10.1093/gigascience/gix030)
Supplement: Reviewer_2_Report_(Original_Submission).pdf [file gix030_Reviewer_2_Report_(Original_Submission).pdf]

Reviewer Report

Title: "Draft genome of the lined seahorse, Hippocampus erectus"

Version: Original Submission **Date:** 12/15/2016

Reviewer name: Christiaan Henkel

Reviewer Comments to Author:

This paper describes the genome assembly and annotation of the lined seahorse, which is intended both as an auxiliary resource accompanying the recently published tiger tail seahorse (ref. 5), and as a first step to support genome-based breeding techniques for aquaculture. The assembly, annotation and validation meet the standards for an Illumina-based genome.

Some minor comments and issues I would like to ask the authors to address:

1) For completeness of the sequencing and assembly process, I would suggest reporting both the Illumina read lengths and the fraction of gaps in the final assembly (which, at 12.8 Mbp, is quite low).

2) On genome size and completeness: the assembly size (458 Mbp) is close to the estimated genome size (489 Mbp), and close to the assembly size of the tiger tail seahorse (502 Mbp). The latter, however, is not close to its estimated size (695 Mbp, line 125), although it is relatively complete based on gene content. Both assemblies contain similar transposon landscapes, with no evidence for a recent burst in activity in the tiger tail seahorse (ref. 5). Therefore, the 695 Mbp estimate might be off? Although it does not impact the current paper, revisiting these estimates for both species might be worthwhile (perhaps using a more sophisticated model, e.g. qb.cshl.edu/genomescope).

3) The BUSCO assessment of assembly completeness by itself does not suggest high quality (line 141), as >25% of orthologues appear to be missing or incomplete. And additional assessment of assembly completeness could establish high quality. For example, ref. 5 includes extensive RNA-seq from the lined seahorse. If these data align very well to the assembly, this would indicate that the BUSCO assessment is an underestimate.

4) On the phylogenetic analysis (lines 206-218): what are 'phase1 sites' (line 212)? I assume this is related to the initial supergene being constructed from translated CDSs, and the final analysis being done on DNA level. This should be explained more clearly.

5) Also, the conclusion (lines 215-218) needs to be reformulated. Currently, it suggests that the distance *between* the seahorses is large, and that therefore they evolve faster than other teleosts (by neutral processes). I do not see this supported by figure 2. The seahorses are closely related, and yes the branch length to other teleosts is long, but this is best explained by the choice of sampled taxons (the seahorses are an early branching group amongst the Percomorpha).

6) On the patristacin genes (line 220-235 and figure 3): interestingly, both seahorse species have six copies. However, figure 3 clearly shows that they do not share all gene duplication (and gene loss) events. For example, the lined seahorse has two copies of the platyfish-like family, which are completely missing in the tiger tail seahorse. In other words (and slightly exaggerated), if the patristacin genes are responsible for or related to male brooding behaviour, then both seahorses evolved parts of this trait independently. (Please note that line 227 should also refer to figure 3 instead of figure 2).

Level of Interest

Please indicate how interesting you found the manuscript: An article whose findings are important to those with closely related research interests

Quality of Written English

Please indicate the quality of language in the manuscript: Needs some language corrections before being published

Declaration of Competing Interests

Please complete a declaration of competing interests, considering the following questions:

- Have you in the past five years received reimbursements, fees, funding, or salary from an organisation that may in any way gain or lose financially from the publication of this manuscript, either now or in the future?
- Do you hold any stocks or shares in an organisation that may in any way gain or lose financially from the publication of this manuscript, either now or in the future?
- Do you hold or are you currently applying for any patents relating to the content of the manuscript?
- Have you received reimbursements, fees, funding, or salary from an organization that holds or has applied for patents relating to the content of the manuscript?
- Do you have any other financial competing interests?
- Do you have any non-financial competing interests in relation to this paper?

If you can answer no to all of the above, write 'I declare that I have no competing interests' below. If your reply is yes to any, please give details below.

I declare that I have no competing interests

I agree to the open peer review policy of the journal. I understand that my name will be included on my report to the authors and, if the manuscript is accepted for publication, my named report including any attachments I upload will be posted on the website along with the authors' responses. I agree for my report to be made available under an Open Access Creative Commons CC-BY license (http://creativecommons.org/licenses/by/4.0/). I understand that any comments which I do not wish to be included in my named report can be included as confidential comments to the editors, which will not be published.

I agree to the open peer review policy of the journal
