# Supplementary material for: Draft genome of the lined seahorse, Hippocampus erectus
Source: Gigascience. 2017 Apr 22;6(6):1–6. doi: 10.1093/gigascience/gix030 (PMC5459928; doi:10.1093/gigascience/gix030)
Supplement: Reviewer_2_Report_(Revision_1).pdf [file gix030_Reviewer_2_Report_(Revision_1).pdf]

Reviewer Report

Title: "Draft genome of the lined seahorse, Hippocampus erectus"

Version: Revision 1 **Date:** 3/13/2017

Reviewer name: Christiaan Henkel

Reviewer Comments to Author:

I am satisfied with most of the answers provided to the issues I raised. In particular, the RNA-seq evidence is useful and convincing. I do think the larger issue of completeness of this genome remains, but not necessarily in the technical sense (i.e. is the assembly of sufficient quality - it is) but in the comparative sense. For example, I do not really agree the number of genes reported is similar to that of the tiger tail seahorse (line 185). Also, I see no explicit support for the claim that the tiger tail seahorse contains a much higher proportion of repeat elements (in answer to my issue 2, on the genome size). But I agree that these issues could be part of a more in-depth comparative study.

That study should then also discuss the patristacin genes in more detail again, as that comparison was dropped from the current manuscript.

Some minor issues:

- I still think the 'phase 1' terminology is confusing (lines 233-237), and the current formulation states the same information twice (?). If correct, I would suggest replacing the entire section ['All these ... (Figure 2)'] with e.g. 'All these nucleotide sequences were concatenated into a supergene for each species, which were used to construct a phylogenetic tree using PhyML (Figure 2).'

- Figure 2 now includes a 'spooted gar'.

Level of Interest

Please indicate how interesting you found the manuscript: An article whose findings are important to those with closely related research interests

Quality of Written English

Please indicate the quality of language in the manuscript: Acceptable

Declaration of Competing Interests

Please complete a declaration of competing interests, considering the following questions:

- Have you in the past five years received reimbursements, fees, funding, or salary from an organisation that may in any way gain or lose financially from the publication of this manuscript, either now or in the future?
- Do you hold any stocks or shares in an organisation that may in any way gain or lose financially from the publication of this manuscript, either now or in the future?
- Do you hold or are you currently applying for any patents relating to the content of the manuscript?
- Have you received reimbursements, fees, funding, or salary from an organization that holds or has applied for patents relating to the content of the manuscript?
- Do you have any other financial competing interests?
- Do you have any non-financial competing interests in relation to this paper?

If you can answer no to all of the above, write 'I declare that I have no competing interests' below. If your reply is yes to any, please give details below.

I declare that I have no competing interests

I agree to the open peer review policy of the journal. I understand that my name will be included on my report to the authors and, if the manuscript is accepted for publication, my named report including any attachments I upload will be posted on the website along with the authors' responses. I agree for my report to be made available under an Open Access Creative Commons CC-BY license (http://creativecommons.org/licenses/by/4.0/). I understand that any comments which I do not wish to be included in my named report can be included as confidential comments to the editors, which will not be published.

I agree to the open peer review policy of the journal
