# Supplementary material for: Draft genome of the lined seahorse, Hippocampus erectus
Source: Gigascience. 2017 Apr 22;6(6):1–6. doi: 10.1093/gigascience/gix030 (PMC5459928; doi:10.1093/gigascience/gix030)
Supplement: Reviewer_3_Report_(Original_Submission).pdf [file gix030_Reviewer_3_Report_(Original_Submission).pdf]

Reviewer Report

Title: "Draft genome of the lined seahorse, Hippocampus erectus"

Version: Original Submission **Date:** 12/29/2016

Reviewer name: Julian Catchen

Reviewer Comments to Author:

In Draft genome of the lined seahorse, Hippocampus erectus, Qiang Lin, et al. present the second seahorse genome and the third genome sequence from the family Syngnathidae for publication as a Data Note. The authors present a nice scaffold-level assembly that will surely be useful in the field. The biggest downside of the paper is that it sometimes reads like it is a supplement to the recently published Hippocampus comes genome from the same first author. The paper needs to provide more details as to some aspects of the assembly, and needs to discuss some of the shortcomings of the analysis (e.g. why was no RNA-seq data generated). Finally, several claims are made in the paper without evidence, apparently borrowed form the H. comes paper, which is inappropriate. These items are detailed below. Finally, the paper needs to be thoroughly copy edited to correct many small language errors.

*On line 86, the authors state that "the lined seahorse is lacking in biological data." This should be made more specific, as the authors cite papers, for example, on the development of transcriptome data for H. erectus, while the H. comes and S. scovelli genomes were just published.

*On line 88, the authors state that "population genetics and molecular adaptability are important research areas for the conservation of this fish species." Why are 'population genetics' and 'molecular adaptability' important?

*In the abstract, the conclusion and on line 95 the authors state that the genome sequence will be further applied for the construction of a high-density genetic linkage map. However, no map is reported in this paper and genome sequence is not required to generate a map. These statements make it appear as if a map has been developed.

*On lines 109-116, the authors discuss error in the are data and provide a list of programs that were applied to clean the data. However, the authors must discuss how many raw reads they started with, how many were discarded or trimmed and from which cleaning process. Further, how many reads contained adaptor sequence and how many were corrected using k-mers. If the numbers are considered abnormally high, the authors should discuss why they had high errors.

*On lines 119-125 the authors discuss how they estimated genome size based on the k-mer spectrum of the raw data, citing the giant panda genome paper for their method. However, the formula they present does not match the formula in the giant panda genome paper. What is the methodological basis for their k-mer estimation method and why was a k-mer size of 17 used (instead of the k-mer size of 27 used for the assembly itself)?

*On line 127 the authors state they used SOAPdenovo2 with 'optimized parameters'. How did the authors come to optimal values for these parameters and what do the parameters listed parenthetically mean?

*On lines 168-176 the authors describe their gene annotation. Why was RNAseq data not used in the annotation (the authors cite a transcriptome for H. erectus)? What are the trade-offs of using H. comes genes to annotate versus using lined seahorse RNA? Would that bias your annotation, particularly with respect to patristacin genes?

*Line 212, what is a "phase1 site" with respect to the coding sequences?

*On line 216-218, the authors state that their tree indicates that "the neutral evolutionary rate of seahorses is significantly higher" than other teleosts. This claim is unsupported. The authors have not presented any data in this paper, based on the tree they constructed to support a claim on neutral evolutionary rates. (This claim originates in the H. comes paper, but the authors applied a number of analytical tests to their specific tree to support that claim.) This claim needs to be better supported with data or removed.

*On lines 226-227, the authors state, "Definitely, we confirmed the existence of six patristacin genes." But how was this done, particularly without the availability of RNAseq data? No method is presented.

*For the species level analysis, the authors employed a maximum likelihood supergene approach with PhyML, but for the phylogenetic analysis of the patristacin genes the authors switch to a Bayesian framework with MrBayes. Why was this done and what method was employed in MrBayes? For example, why not use a partitioned gene-oriented approach with PhyML in this case? Some additional description of what went into the phylogenetic analyses should be presented. How did you decide on

the orthologous genes to include, why not include outgroup genes and outgroup species in the gene tree? Why not apply and compare both methods for the species and gene trees as is frequently done?

*The authors should consider including the S. scovelli orthologs in their species level analysis.

*On lines 228-235, the authors state the that the expansion of patristacin genes is related to platyfish and elements of male pregnancy. However, these claims are presented without any evidence, but simply a citation back to the H. comes genome paper. All the authors have presented is an unrooted tree showing a number of teleost patristacin genes grouped in different clades. The individual gene family members are not labeled and cannot be compared among the various orthologs and branches on the tree. This claim needs to be better supported with data or removed.

*The authors should consider including the patristacin genes from the newly published Sygnathus scovelli in their phylogenetic analysis.

*Where can the assembled genome and annotation be obtained from? Only the raw data appear to have been deposited in NCBI.

Minor errors:

Line 75-75, the meaning of "seasonal migration conflicting their small home range" is not clear.

Line 210, 213, "Perl" should be properly capitalized.

Line 225, "blasted" is not a verb.

Level of Interest

Please indicate how interesting you found the manuscript: An article of importance in its field

Quality of Written English

Please indicate the quality of language in the manuscript: Needs some language corrections before being published

Declaration of Competing Interests

Please complete a declaration of competing interests, considering the following questions:

- Have you in the past five years received reimbursements, fees, funding, or salary from an organisation that may in any way gain or lose financially from the publication of this manuscript, either now or in the future?
- Do you hold any stocks or shares in an organisation that may in any way gain or lose financially from the publication of this manuscript, either now or in the future?
- Do you hold or are you currently applying for any patents relating to the content of the manuscript?
- Have you received reimbursements, fees, funding, or salary from an organization that holds or has applied for patents relating to the content of the manuscript?
- Do you have any other financial competing interests?
- Do you have any non-financial competing interests in relation to this paper?

If you can answer no to all of the above, write 'I declare that I have no competing interests' below. If your reply is yes to any, please give details below.

I have no competing interests.

I agree to the open peer review policy of the journal. I understand that my name will be included on my report to the authors and, if the manuscript is accepted for publication, my named report including any attachments I upload will be posted on the website along with the authors' responses. I agree for my report to be made available under an Open Access Creative Commons CC-BY license (http://creativecommons.org/licenses/by/4.0/). I understand that any comments which I do not wish to be included in my named report can be included as confidential comments to the editors, which will not be published.

I agree to the open peer review policy of the journal
